# Requirement of Fc-Fc Gamma Receptor Interaction for Antibody-Based Protection against Emerging Virus Infections

**DOI:** 10.3390/v13061037

**Published:** 2021-05-31

**Authors:** Shamus P. Keeler, Julie M. Fox

**Affiliations:** 1 Division of Pulmonary and Critical Care Medicine, Washington University School of Medicine, St. Louis, MO 63110, USA; skeeler@wustl.edu; 2 Laboratory of Viral Diseases, National Institute of Allergy and Infectious Diseases, National Institutes of Health, Bethesda, MD 20892, USA

**Keywords:** antibodies, Fc effector functions, emerging viruses

## Abstract

Identification of therapeutics against emerging and re-emerging viruses remains a continued priority that is only reinforced by the recent SARS-CoV-2 pandemic. Advances in monoclonal antibody (mAb) isolation, characterization, and production make it a viable option for rapid treatment development. While mAbs are traditionally screened and selected based on potency of neutralization in vitro, it is clear that additional factors contribute to the in vivo efficacy of a mAb beyond viral neutralization. These factors include interactions with Fc receptors (FcRs) and complement that can enhance neutralization, clearance of infected cells, opsonization of virions, and modulation of the innate and adaptive immune response. In this review, we discuss recent studies, primarily using mouse models, that identified a role for Fc-FcγR interactions for optimal antibody-based protection against emerging and re-emerging virus infections.

## 1. Introduction

Emerging viral infections are caused by newly discovered viruses or viruses that are increasing in incidence or geographical range. These infections cause significant morbidity and mortality, and can have additional economic and societal costs. Within the past 15 years, the world has experienced 2 pandemics, the 2009 H1N1 and the Coronavirus Disease 2019 (COVID-19), and several large-scale epidemics that had global consequences, including the 2014–2016 Ebola outbreak and the 2015–2016 Zika virus epidemic [1,2,3,4]. To date, the COVID-19 pandemic has resulted in more than 3 million deaths and has cost the global community trillions of dollars in both response costs and economic output [5]. The need for therapeutics that are either broad in spectrum or that can be developed rapidly after discovery of an emerging virus is paramount. Monoclonal antibodies (mAbs) are an effective treatment option for viral infections, and recent advances in development and production make mAbs a key therapeutic strategy for emerging viral infections.

**Figure 1:**
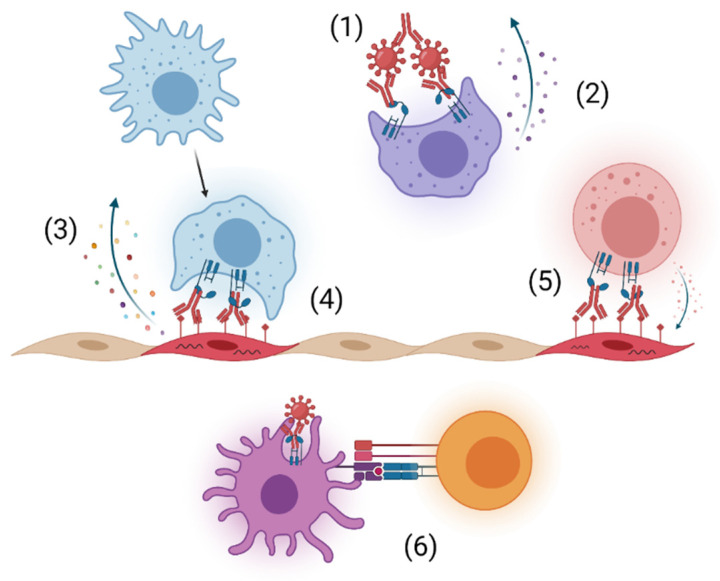
Potential Fc-FcγR interactions for enhanced protection with mAb administration. (**1**) Opsonization of viral-antibody immune complexes and clearance by phagocytic cells. (**2**) Altered activation and pro-inflammatory response from phagocytic cell. (**3**) Secretion of chemokines and cytokines to promote cellular recruitment and modify inflammation. (**4**) Clearance of infected cells through antibody-dependent cellular phagocytosis (ADCP) by phagocytic cells. (**5**) Removal of infected cells through antibody-dependent cellular cytotoxicity (ADCC) by effector cells. (**6**) Enhanced T cell activation through Fc-FcγR mediated DC maturation. Created with BioRender.com.

Viral infection results in the generation of anti-viral antibodies that limit infection and aid in clearance during acute disease and subsequently prevent reinfection. Antibodies consist of two heavy and two light chains that form two distinct regions, the fragment antigen binding (Fab) region, which contains the variable region that binds to the antigen, and the fragment crystallizable (Fc) region, which interacts with host proteins, such as Fc receptors and complement, to facilitate effector functions [6]. The most abundant immunoglobulin isotype in plasma is IgG, and it is widely studied and used in the clinic for its long half-life, neutralizing potency, and functional activities [7]. IgG can neutralize infection by directly blocking the viral replication cycle, form immune complexes that enhance phagocytosis through opsonization, and activate immune cells through Fc-Fc gamma receptor (FcγR) interactions (Figure 1). Additionally, interaction of FcγRs on immune cells with the Fc region of antibodies bound to viral proteins on the surface of infected cells can trigger clearance of infected cells through antibody-dependent cellular cytotoxicity (ADCC) and antibody-dependent cellular phagocytosis (ADCP) (Figure 1) [6]. During some viral infections, Fc-FcγR interactions assist in alternative pathways of virion entry that can enhance infection in FcγR-bearing cells and altered host response, resulting in antibody-dependent enhancement (ADE), although this is not a main focus of this review [8,9].

Numerous viral models, including influenza, coronaviruses, alphaviruses, HIV, filoviruses, and flaviviruses, demonstrated that antibodies are more effective in vivo with Fc-mediated effector functions [10,11,12,13,14,15]. Enhanced cellular activity through Fc-FcγR interaction can rapidly clear infections but may result in increased immune-mediated pathogenesis. A balance between these outcomes is necessary for optimal antibody-based protection. Emerging viral infections are a significant public health threat, and optimizing antibody-based protection through balanced engagement of Fc-mediated functions will result in more effective therapeutics. Here, we review studies that evaluated the necessity of Fc-FcγR interactions for mAb-based therapies, primarily using mouse models, during emerging and re-emerging virus infections.

## 2. Approaches to Study Fc Effector Functions In Vivo

The requirement of Fc effector functions for IgG-mediated protection in vivo can be evaluated using a combination of IgG subclass switching, recombinant mAb variants to enhance or eliminate FcγR interactions, genetic knockout mice, and transgenic FcγR humanized mice. These reagents provide a robust toolkit for experiments focused on the role of FcγR-mediated protection. The following sections introduce these reagents focusing on the techniques discussed later.

### 2.1. IgG Subclass and FcγRs

The subclass of IgG impacts the affinity to specific mouse FcγRs (Table 1). There are four subclasses of mouse IgGs, including IgG1, IgG2a/c, IgG2b, and IgG3 and four mouse FcγRs, including FcγRI, FcγRIIb, FcγRIII, and FcγRIV. The activating FcγRs (FcγRI, FcγRIII, and FcγRIV) associate with the Fc common gamma chain that contains an immunoreceptor tyrosine-based activation motif (ITAM), which is phosphorylated by Src family protein tyrosine kinases (PTKs) following binding and results in activation [16]. The single inhibitory receptor, FcγRIIb, contains an immunoreceptor tyrosine-based inhibitory motif (ITIM), which is also phosphorylated by Src PTKs but binds to tyrosine phosphatase that dephosphorylate proteins in the activating pathways [17]. IgG2a/c binds to all FcγRs and to FcγRI and FcγRIV with the highest affinity [18]. IgG2b binds to all FcγRs, while IgG1 binds with the highest affinity to FcγRIIb and to FcγRIII with lower affinity [18,19]. IgG3 has minimal interaction with FcγRs [18,20,21]. For these reasons, the IgG2a/c isotype is considered the most functional since it engages all activating FcγRs, while IgG1 is the least functional by preferentially interacting with the inhibitory FcγRIIb [20]. The expression of FcγRs varies across cell types. Mouse neutrophils express FcγRs IIb, III, and IV; NK cells only express FcγRIII; B cells only express FcγRIIb; and dendritic cells (DCs), macrophages, and monocytes express all FcγRs [19].

In the clinic, the use of human or humanized mAbs is preferred to extend antibody half-life and prevent the development of anti-drug antibodies; however, most therapeutic antibodies are initially tested in mouse models. There are four human IgGs (IgG1, IgG2, IgG3, and IgG4) with variable affinity for mouse FcγRs (Table 1). Human IgG1, IgG3, and IgG4 bind to all mouse FcγRs with IgG3 having the highest relative affinity; however, human IgG1 is more potent at inducing ADCP and ADCC in mouse and human macrophages, NK cells, and polymorphonuclear leukocytes [22,23]. Therefore, in mice, mouse IgG2a/c and human IgG1 are the most effective inducers of antibody effector functions through interactions with the activating FcγRs, and subclass switch of mAbs can be used to test functional activity in vivo.

### 2.2. Fc Mutations

Modifications in the Fc region of antibodies through elimination of the N-linked glycosylation site or introduction of other mutations on the heavy chain have been shown to alter Fc effector functions and was recently extensively reviewed [24]. For the purposes of this review, we will discuss the mutations used in the described studies. Removal of the N-linked glycosylation site on the heavy chain with an N297Q or N297A mutation reduces Ab interactions with FcγRs and C1q for human and mouse Ab [25,26]. Although aglycosylated mAbs can be prone to aggregation, they have a similar half-life to the intact mAbs [27,28]. Alternatives that maintain a glycosylated mAb and abrogate binding to all FcγRs include D265A or G236R L328R (GRLR) [29,30]. The L234A L235A (LALA) mutation reduces binding to activating FcγRs and C1q. The addition of the P329G mutation (LALA-PG) eliminates FcγR or C1q interaction [31,32,33]. Fc-C1q binding can be targeted using the K332A mutation (KA), which results in a slight reduction (<2 fold) in FcγR binding affinity but a complete loss of C1q binding [31]. Alternatively, introduction of the mutations G236A/S239D/A330L/I332E (GASDALIE) increases Fc affinity for human FcyRIIIa, G236A/A330L/I332E (GAALIE) increases affinity to human FcγRs IIa and IIIa, and G236A (GA) enhances affinity to human FcγRIIa, which results in increased effector functions in vivo using transgenic human FcγR mice [34,35,36]. These approaches are useful for testing Fc effector function of mAbs in specialized transgenic mouse models or if access to FcγR-specific knockout mice, as described below, is not readily available.

### 2.3. Knockout and Transgenic Mice

Knockout mice and transgenic mice expressing the human FcγRs can be used to identify specific FcγRs that are critical for protection, delineating the role of Fc-complement versus Fc-FcγR interactions, and determining the functional activity of a mAb in the context of human immunity. The most commonly used mouse model lacks the Fc common gamma chain (FcRγ^−/−^), which is deficient in the activating FcγRs and the Fc epsilon receptor I (FcεRI) [37]. Alternatively, a FcRα null mouse was generated that lacks the α-chains for all of the FcγRs, thus maintaining a functional Fc common gamma chain and FcεRI [38]. Mice deficient in individual FcγRs have been produced as well as double knockouts of FcγRI and FcγRIII [39,40,41,42,43]. While useful, single knockouts may influence the expression of the remaining FcγRs [41]. The expression pattern of FcγRs differs in human and mouse immune cells, which complicates the extrapolation of data generated in mouse studies and contributes to discrepancies observed when comparing these studies to those performed in humans [44]. A transgenic FcγR humanized mouse was generated on the background of the FcRα null mice and expressed the cellular patterns of human FcγRs on the appropriate cell type, providing an opportunity to study the potential outcomes of administering human IgG in the clinic [38].

## 3. Optimal mAb Efficacy In Vivo through Fc-Effector Functions

### 3.1. Influenza Virus

Influenza viruses are enveloped viruses with a segmented, single-stranded, negative sense RNA genome in the *Orthomyxoviridae* family. Influenza A and B viruses cause seasonal, yearly outbreaks with the potential threat of global pandemics. The most recent pandemic occurred in 2009 with the emergence of a novel swine-origin H1N1 influenza virus [45]. The two surface glycoproteins, hemagglutinin (HA) and neuraminidase (NA), are involved in binding to sialic acids for cellular attachment and fusion and virus release through sialic acid cleavage, respectively, and are the main targets of the neutralizing antibody response [46]. There are 18 HA and 11 NA subtypes that have been identified in humans, birds, and bats, with the majority of human infections being attributed to two subtypes, H1N1 and H3N2, which are the currently circulating seasonal influenza strains [46]. The HA is displayed as a homotrimer with a variable head domain that interacts with sialic acid and a more conserved stalk region that is involved in membrane fusion [47]. The NA is a homotetramer with a catalytic head domain that is involved in sialic acid cleavage and a stalk region that is variable in length between NA subtypes [46]. In infected cells, the HA and NA are transported to the cellular plasma membrane for virion budding. The ion channel, matrix 2 protein (M2), is also present on the cell surface and is incorporated into the virion, where it aids in viral entry [48]. The most abundant protein on the virion is HA, followed by NA with minimal amounts of M2 [49]. Since the viral proteins are expressed on the cellular surface, reactive antibodies can bind and remove infected cells through FcγR or complement interaction in addition to virus neutralization and clearance.

The identification of broadly reactive antibodies has been a cornerstone in the effort to develop a universal influenza vaccine by targeting conserved regions on viral proteins, such as the HA stalk and M2 ectodomain (M2e). Numerous studies have highlighted the necessity of Fc-FcγR interaction for protection against multiple influenza subtypes using broadly neutralizing and non-neutralizing, cross-reactive antibodies targeting the HA head, HA stalk, NA, and the M2e. This stems from an early study in which mice lacking the Fc common gamma chain were shown to be more susceptible to a lethal influenza infection, even though the FcRγ^−/−^ mice develop similar antibody titers as wild type (WT) mice indicating that factors outside of antibody neutralization were important for protection [10].

Cross-reactive and broadly neutralizing anti-HA antibodies predominantly protect in vivo against multiple HA subtypes through Fc-FcγR interactions. Broadly neutralizing anti-H1 mAbs targeting the HA stalk required Fc-FcγR interactions to protect from a lethal H1N1 infection [50]. To evaluate Fc effector functions, the authors isotype switched one of the mAbs, 6F12, from a mouse IgG2a to a mouse IgG1 and compared these to a D265A variant [50]. The IgG2a mAb reduced weight loss, conferred 100% survival, and reduced viral load in the lungs, while the IgG1 and D265A variants were similar to PBS-treated mice [50]. The IgG2a mAb lost its efficacy in FcRγ^−/−^ mice, and analysis of single FcγR knockout mice determined that protection was primarily mediated through FcγRIV, but FcγRI and III can partially compensate in the absence of FcγRIV [50]. In contrast, the IgG subclass did not impact the protective efficacy of H1 strain-specific mAbs targeting the HA head, thus suggesting Fc-FcγR interaction is not essential for strain-specific mAb protection [50]. Other pan-H1 mAbs, including neutralizing and non-neutralizing mAbs, that target the HA head domain reduced weight loss and prevented mortality, which was lost with the D265A mutation [51].

Non-neutralizing and neutralizing, broadly reactive anti-H4 mAbs showed ADCC activity in vitro and increased survival during a lethal H4N6 challenge [52]. Although the majority of the anti-H4 antibodies that were protective in vivo also neutralized the virus, it is likely that antibody effector functions enhanced protection. Similarly, broadly reactive anti-H7 mAbs, which either neutralized the virus and targeted the H7 stalk or non-neutralizing mAbs that bound outside of traditional sites, protected against a lethal influenza challenge [53]. However, the neutralizing mAbs cleared the virus faster in the lungs than the non-neutralizing mAbs at 6 days post-infection (dpi) [53]. Mice treated with D265A variants of the broadly reactive, non-neutralizing anti-H7 mAbs showed significant weight loss compared to the intact mAb, but the D265A variants of a neutralizing mAb still were protective [53]. In vitro effector function assays identified that the neutralizing anti-H7 mAbs highly induced ADCC and phagocytosis while the non-neutralizing mAbs only enhanced phagocytosis [53]. The authors determined that the non-neutralizing mAbs accelerated the endogenous anti-H7 antibody response, most likely though phagocytosis of immune complexes, which aided in in vivo protection [53].

FI6 and an optimized variant of FI6 (FI6v3) broadly neutralize across group 1, including subtypes H1 and H5, and group 2, including subtypes H3, H4, and H7, influenza viruses [54]. Administration of either mAb increased survival following challenge with various subtypes of influenza, including H1N1, H3N2, H5N1, in mice and ferrets [54]. The FI6-LALA variant dramatically reduced protective efficacy as compared to the intact mAb or an mAb with a KA mutation [54]. Similarly, another group 1 and 2 broadly neutralizing mAb, 2G02 completely prevented mortality and weight loss following an H1N1 challenge, while the D265A variant mirrored the isotype treated mice [51].

Multiple reports have identified specific immune-cell populations that contribute to the Fc-mediated protection during influenza A infection with anti-HA mAb treatment (Table 2). In in vitro studies, broadly reactive antibodies from human and macaque samples activated NK cells, as determined by increased expression of CD107a and IFNγ, and the activated NK cells eliminated infected cells, suggesting ADCC as a mechanism to remove infected cells [55]. Other studies identified macrophages as the key cell type for mAb-mediated protection. Huber et al. showed increased uptake of labeled influenza virus in a macrophage cell line in the presence of influenza immune serum compared to naïve serum [10]. Depletion of alveolar macrophages in vivo using clodronate liposomes reduced protection from weight loss, death, and viral load in the lungs at 6 dpi after homologous influenza challenge when administered broadly reactive non-neutralizing antibodies, while the presence or absence of alveolar macrophages minimally impacted the outcome with neutralizing mAb treatment [56]. Alveolar macrophages were also required for protection with non-neutralizing, broadly reactive mAbs during heterologous influenza virus challenge [56]. Adoptive transfer of alveolar macrophages into GM-CSF knockout mice rescued the efficacy of a non-neutralizing mAb [56]. Furthermore, the non-neutralizing mAbs induced higher levels of pro-inflammatory cytokines, such as TNFα, IL-6, MCP-1, IL-12p40, and G-CSF, in the airways following influenza infection, which correlated with increased inflammation [56]. However, both neutralizing and non-neutralizing broadly reactive mAbs induced an inflammatory signature in an Fc-dependent manner when incubated with primary alveolar macrophages in vitro [56]. Using a transgenic humanized FcγR mouse model, Fc interaction of the GAALIE or GA variant of the broadly neutralizing mAb, FI6v3 with human FcγRs IIA and IIIA on dendritic cells (DCs) enhanced DC maturation, which augmented the CD8^+^ T cell response, enhanced survival, and reduced weight loss [35]. These studies highlight that multiple mechanisms and cell types involving Fc-FcγR interactions can modify the immune response and aid in protection for mAbs targeting the influenza A HA protein.

Broadly reactive antibodies specific to the HA head of influenza B enhanced survival and reduced weight loss following a lethal challenge [62]. While these antibodies could neutralize the virus in vitro, the mAbs also showed ADCC activity in vitro [62]. For influenza B, FcγR interaction is not specific to HA head antibodies, but rather a function of most broadly reactive antibodies. This includes non-neutralizing, broadly reactive anti-influenza B mAbs targeting the HA stalk region. When these mAbs were sorted based on isotype, the mouse IgG2a mAbs were the most effective at reducing weight loss followed by mouse IgG2b then mouse IgG1, which correlated with increased ADCC activity in vitro [63]. Furthermore, the authors showed that beyond the breadth of binding, mAbs recognizing conformational epitopes induced more ADCC activity and reduced weight loss in vivo compared to mAbs with linear epitopes [63]. This has also been observed for anti-NA antibodies. An NA-based recombinant protein vaccine induced antibodies that inhibit NA activity and protect mice from homologous and heterologous challenge [64]. Passive transfer of the NA-immune serum to FcRγ^−/−^ mice showed reduced efficacy with an increase in weight loss following homologous virus challenge [64]. A functional Fc region was required to increase survival for a pan-N1 mAb, 3C05; however, introduction of the D265A mutation into a strain specific anti-N1 mAb, 3C02, did not impact in vivo protection [51]. Contrary to this study, a panel of H1N1 specific anti-NA antibodies that bound to the lateral surface of NA head and had varying NA inhibitory (NAI) activity either partially or completely relied on Fc effector functions to prevent weight loss and enhance survival [65]. While the mAbs with the highest NAI activity were also the most potent activator of ADCC and ADCP, using an in vitro reporter assay, the N297Q variants partially protected mice from lethal challenge [65]. The mAbs with little to no NAI activity had reduced ADCC and ADCP activity in vitro, but required FcγR-mediated functions to prevent lethality [65]. 

Outside of the two main surface glycoproteins, Fc effector functions enhance efficacy of antibodies targeting the influenza M2 protein. Multiple studies showed passive transfer of mAbs targeting the M2e or M2e vaccination schemes reduce weight loss and viral burden in the lungs and increase survival [66,67,68,69]. While the M2 protein is inserted into the virion, it is also abundantly expressed on the surface of virally infected cells to which anti-M2 antibodies can bind [48]. A study compared two mAbs that bound a similar epitope on M2e. One mAb, mAb 37, was mouse IgG1, and the other, mAb 65, was mouse IgG2a [68]. Using a combination of knockout mice, the authors showed both mAbs required FcγRs to provide 100% survival against an H3N2 challenge; however, the IgG1 mAb utilized predominantly FcγRIII and partially FcγRI, while FcγRIV contributed to the survival observed with the IgG2a mAb [68]. Interestingly, the reduction in viral burden associated with the IgG2a mAb partially relied on FcγRs I and III [68]. When challenged with other H3N2 strains, the IgG2a mAb reduced viral titers in the lungs and weight loss in an FcγRI and FcγRIII dependent fashion [43]. Combining the idea that antibodies targeting M2e need to interact with FcγRs for optimal activity, a bi-specific antibody was developed consisting of two single domain antibodies with one variable region specific for the M2e and the other variable region binding FcγRIV [70]. Influenza-challenged mice treated intranasally with the bispecific fusion construct showed increased survival that was FcγRIV dependent [70].

Vaccines targeting the M2e have addressed the necessity of Fc-FcγR interaction for optimal protection. FcRγ^−/−^ mice immunized with a virus-like particle that expressed repeats of M2e failed to prevent weight loss and death and reduce viral titers in the lung following challenge when compared to vaccinated WT mice [67]. The FcRγ^−/−^ mice induced a similar antibody response as the WT mice; however, the absence of FcRγ signaling increased IL-6 expression and numbers of T cells expressing IFN-γ and IL-4 in the lungs [67]. This suggests that the lack of clearance of infected cells and virus particles or signaling through other innate cells through FcγRs resulted in increased inflammation. A vaccine designed with the M2e coupled to the hepatitis B core (HBc) protein induced antibodies that bound to infected cells, mediated protection, and reduced viral burden at multiple time points post-challenge [57]. The vaccine showed reduced efficacy when NK and NKT cells were depleted using anti-asialo-GM1 [57]. A similar study also observed that passive transfer of immune serum using an M2e-HBc vaccine platform required FcγRs, predominantly mouse FcγRI and FcγRIII, to reduce weight loss and increase survival [58]. However, alveolar macrophages were necessary to provide complete protection following influenza challenge, which was determined by depletion of alveolar macrophages by intratracheal clodronate liposome treatment of WT mice or through adoptive transfer of wild type alveolar macrophages into mice lacking FcγRI and FcγRIII [58]. Some possibilities for this discrepancy could be the location and frequency of vaccination, the addition of an adjuvant in the vaccine formulation, and the method of depletion.

Influenza virus remains a continuing concern with an estimated 35.5 million symptomatic cases and over 34,000 deaths during the 2018–2019 season, and this does not include the potential emergence of novel subtype variants [71]. The described studies highlight the variable requirement of Fc-FcγR interactions for mAb therapy during influenza virus infection. Factors such as viral protein target, breadth of reactivity, vaccination scheme, and neutralization potency can influence the use of Fc-effector functions and the cell type specific for protection. While most studies administer mAbs through intraperitoneal injections, the utility of administering mAbs intranasal should be considered since influenza primarily infects the respiratory tract. In a recent study, Vigil et al. evaluated delivery methods of pan-group 1, pan-group 2, and influenza B mAbs, and identified that Fc-mediated protection was specific to intraperitoneal administration compared to intranasal delivery, which depended more on neutralization [72]. However, intranasal administration of a mouse IgG1 anti-N1 mAb, N1-C4, in FcRγ^−/−^ mice still reduced weight loss despite no reduction in viral burden in the lungs compared to WT mice [73]. While intranasal administration of mAbs is not currently approved, it could be a viable option to provide local protection. As more mAbs are identified and characterized, a clearer picture is emerging that specific antibody features are universal to mAbs requiring Fc effector functions.

### 3.2. Coronaviruses

Viruses within the family *Coronaviridae* have a single-stranded, positive sense RNA genome and infect a wide range of hosts, including humans. Several strains of human coronaviruses (HuCoVs) are endemic and cause mild upper respiratory infections as part of a cluster of “common cold” viruses. Within the past 20 years, 3 highly pathogenic HuCoVs have emerged, causing regional and global outbreaks, and most recently the COVID-19 global pandemic. The pathogenic HuCoVs are severe acute respiratory syndrome coronavirus (SARS-CoV), Middle East respiratory syndrome-related coronavirus (MERS-CoV), and severe acute respiratory syndrome coronavirus 2 (SARS-CoV-2). Antibody-based therapies have been pursued for all three pathogenic HuCoVs, but research into Fc-mediated effector functions has been limited to SARS-CoV and SARS-CoV-2 with most focused on the latter. Multiple mAbs targeting the spike (S) glycoprotein of MERS-CoV have been isolated and demonstrated to have in vivo efficacy with both prophylactic and therapeutic treatment, but the role of Fc effector functions has not been evaluated to date [74,75,76,77].

Both SARS-CoV and SARS-CoV-2 bind to the entry receptor angiotensin-converting enzyme 2 (ACE2) using the trimeric S glycoprotein [78,79]. The S protein is composed of two subunits: S1, which contains an N terminal domain and the receptor binding domain (RBD) responsible for recognition of ACE2, and the C terminal S2, which is involved in membrane fusion [80]. Due to the role of the RBD in recognition of the host receptor, the majority of neutralizing mAbs being studied are reactive to this subunit but cognizant of the risk of rapid viral escape; recent studies have identified mAbs targeting other subunits, including the N terminal domain [11,59,81,82,83,84,85]. The anti-SARS-CoV-2 human mAbs C104 and C110 reduced viral burden in mice, but the introduction of the GRLR mutation significantly reduced their in vivo efficacy as measured by lung viral loads [82]. The authors confirmed these results by grafting the variable domains of C104 onto a mouse IgG1, IgG2a, and IgG1-D265A variant [82]. While these murinized antibodies had similar neutralization potencies, both C104-IgG1 and C104-IgG_D265A_ were significantly less protective in vivo, and only the C104-IgG2a demonstrated comparable potency to its human counterpart [82]. These results highlight the importance of Fc-mediated effector function for optimal protection against SARS-CoV-2 but also indicate potential cell mediators of this protection as the mouse IgG2a binds the FcγRIV on monocytes, neutrophils, and dendritic cells [86]. Administration of the LALA variant of the anti-RBD antibody SC31 resulted in increased weight loss and mortality in SARS-CoV-2 infected K18-human ACE2 transgenic mice compared to treatment with the parental antibody [85]. Mice treated with either SC31 or SC31-LALA showed similar viral loads and levels of proinflammatory cytokines, but SC31 treated animals had reduced levels of the chemokines CXCL10 and CCL2 [85]. Similar results were observed with antibodies COV2-2676 and COV2-2489, which target the N-terminal domain of the S1 subunit [87]. Treatment with either COV2-2676-LALA and COV2-2489-LALA resulted in increased weight loss and lung pathology compared to mice treated with the intact variants [87]. Winkler et al. (2021) performed analogous experiments comparing the efficacy of LALA variant antibodies in K18-human ACE2 transgenic mice and observed a similar loss of potency compared to intact antibodies, and extended these findings with COV2-2050 in an additional animal model and depletion studies in mice. SARS-CoV-2 infected Syrian hamsters treated with the anti-RBD antibody COV2-2050 one day after infection resulted in a reduction in weight loss, viral loads, and markers of inflammation compared to control animals, but the benefits of the antibody treatment were lost when hamsters were treated with the LALA variant [11]. Antibody depletion of Ly6C^hi^ monocytes but not NK cells or neutrophils reduced the efficacy of the intact COV2-2050 antibody when given therapeutically [11]. The Fc effector functions were found to be dispensable when the antibodies were administered prophylactically [11]. The monocyte-depleted mice treated with the intact antibody had similar viral RNA levels as non-depleted mice, indicating no role for monocytes in viral clearance, but the depleted mice had increased weight loss and lung pathology [11]. Additionally, Fc engagement resulted in improved respiratory function, and transcriptional profiling showed down regulation of innate immune signaling and upregulation of tissue repair [11]. CCR2^+^ monocytes differentiate into interstitial macrophages and monocyte-derived dendritic cells, and these cells are known to be critical mediators of the inflammatory response to viral infections [88,89,90]. Earlier work with SARS-CoV corroborates this role for monocytes and monocyte-derived cells in reducing coronavirus disease through Fc effector function engagement. The efficacy of SARS-CoV antiserum was reduced in mice depleted of monocytes and neutrophils and partially in mice depleted of macrophages using clodronate [59]. The depletion of complement, NK cells or neutrophils had no influence on the efficacy of SARS-CoV antiserum treatment [59]. 

The use of passively transferred immunity via mAbs or convalescent serum for the treatment of HuCoVs was tempered in the past due to concerns about ADE of infections. These concerns arose due to reports of ADE in feline coronaviruses and increased immunopathology observed during homologous challenge of SARS-CoV vaccinated mice and non-human primates [84,91,92,93,94,95]. Out of an abundance of caution, some scientists only pursued and tested mAbs with abrogated Fc effector functions, which was the case with the anti-SARS-CoV-2 antibody C86 [96]. These concerns about ADE in the known HuCoVs have proven to be unwarranted with the majority of studies in animals and humans demonstrating no risk for enhanced disease due to passively transferred immunity. In fact, the summarized data highlight the importance of Fc effector functions in the treatment of HuCoVs. When administered therapeutically, anti-SARS-CoV-2 mAbs with intact Fc regions provided greater protection and reduced disease burden compared to their Fc-null counterparts. Monocyte and monocyte-derived cells are necessary cell mediators of this protection, potentially through reprograming of the immune system away from a prolonged inflammatory response and towards tissue repair and homeostasis. Recent studies comparing the antibody repertoires in patients with mild-to-moderate COVID-19 to critically ill patients further bolster these conclusions. The serum from convalescent patients skewed towards complement activity and ADCP responses compared to more dominant pro-inflammatory and ADCC responses observed in patients that eventually died of COVID-19 [97,98]. The current understanding of the role of FcγR in HuCoVs is still limited, and much is left to be discovered. In particular, it is unknown what effector molecules are most critical for mAb therapeutic activity, and no work has been done to delineate the importance of specific FcγRs.

### 3.3. Alphaviruses

Alphaviruses are mosquito-borne enveloped viruses with a single-stranded, positive sense RNA genome. Alphaviruses that infect humans are generally categorized into the arthritogenic or encephalitic based on the clinical manifestations. Arthritogenic alphaviruses, including chikungunya (CHIKV), Mayaro (MAYV), and Ross River virus (RRV), cause severe polyarthritis and polyarthralgia with mortality being rare, while the encephalitic alphaviruses, including Venezuelan equine encephalitis (VEEV), eastern equine encephalitis (EEEV), and western equine encephalitis viruses (WEEV), can progress to CNS infection and encephalitis, resulting in fatalities and long-term neurological sequalae [99]. Chikungunya virus has spread across the globe with the most recent epidemic in the Caribbean and South America in 2013–2014 [100]. VEEV has caused local outbreaks in Central and South America and the other encephalitic viruses produce sporadic cases in the Americas, but the encephalitic viruses are classified as category B bioterrorism agents due to potential aerosol infection [101]. Even with the continued emergence and re-emergence of these viruses, there are no approved treatments or vaccines.

The majority of studies directly addressing Fc effector functions have been limited to antibodies against arthritogenic alphavirus. Alphaviruses attach and enter cells through binding of the surface glycoproteins E2 and E1 [102,103]. The E2 and E1 glycoproteins form a heterodimer, with E2 protein positioned above E1. The heterodimer is arranged as trimers on the virions and is the main target of neutralizing antibodies [104,105]. The E2 protein contains three domains: domain A, which is centrally located; domain B, located distal to the virion; domain C, which is proximal to the virion surface [105]. The E2 domain A and B are the dominant targets of neutralizing antibodies with broadly neutralizing antibodies targeting the B domain [106,107]. In infected cells, the E2 and E1 heterodimer is transported to the plasma membrane and studs the surface of the cells in preparation of virion budding. Anti-CHIKV mAbs bound to the glycoproteins on the surface of CHIKV-infected cells prevent budding of virions and promote clustering of replication spherules, which enhanced Fc-FcγR engagement as shown using reporter cells expressing human FcγRIIIa [108]. The N297Q variant of the antibodies prevented the activation of FcγR-bearing cells [108]. These results correlate with in vivo studies using other anti-alphavirus mAbs.

Administration of the N297Q variant of the potently neutralizing anti-CHIKV mAb, CHK-152, resulted in increased mortality and disease score compared to the intact mAb [107]. In a follow-up study using a combination of two neutralizing anti-CHIKV mAbs that bind the E2 or E1 protein, CHK-152 and CHK-166, interaction of the Fc region of the mAbs with FcγRs enhanced clearance of infected cells and reduced foot swelling (i.e., clinical disease) when administered 3 days after infection compared to N297Q mAb variants. These results were confirmed in FcRγ^−/−^ mice [12]. Interestingly, the day after intact mAb administration, there was an influx of immune cells, specifically an increase in the number of CD45^+^ cells, monocytes, and neutrophils, at the site of infection compared to N297Q or isotype-treated mice [12]. This correlated with increased levels of CCL2, CCL3, CCL4, and CCL5. At 7 dpi, there were reduced levels of chemokines with the intact mAb treatment [12]. Through a series of antibody depletion studies, mAb interaction with monocytes was identified to be required to reduce viral burden and clinical disease [12]. An additional study using anti-CHIKV mAbs, CHK-124 and CHK-263, showed that FcγR engagement was required to reduce viral RNA for CHK-124 but not CHK-263, and Fc-FcγR interaction did not impact clinical disease for either mAb [109]. The difference between the mAbs could be related to the epitope or angle of binding since CHK-124 bound exclusively to the B domain of E2 and CHK-263 had a larger footprint binding to the B domain and β-linker of E2 and domain II of E1 [109]. The lack of Fc-FcγR dependent decrease in clinical disease could be attributed to other factors such as time of administration and dose.

While early administration of anti-CHIKV mAbs can limit disease in the absence of Fc effector functions, mAbs against MAYV required a functional Fc region to protect against mortality when administered prior to infection [110]. Using a panel of mouse anti-MAYV mAbs that primarily bound to the B domain of E2, mAbs of an IgG2a subtype prevented foot swelling and reduced viral RNA, while IgG1 mAbs failed to prevent mortality and only partially reduced foot swelling [110]. When anti-MAYV mAbs were isotype-switched from a highly functional mouse IgG2a to a low functional mouse IgG1 or a human IgG1 N297Q variant, there was a significant drop in survival and mild reduction in foot swelling [110]. During RRV infection, administration of a broadly neutralizing anti-alphavirus mAb, CHK-265, or the N297Q variant 1 day before infection decreased viral RNA burden in local and systemic tissues at early time points post-infection [111]. Another broadly neutralizing mAb, RRV-12, did not require Fc-FcγR interaction either for protection, as the LALA variant reduced viral burden to a similar level as the intact mAb when administered 1 dpi [112].

Across the arthritogenic alphaviruses, the antibody epitope, dose, and timing of mAb administration may not universally dictate the necessity of Fc-FcγR interaction, but rather these factors are largely influenced by the virus. Viral tropism, conformation of glycoproteins on the cellular surface, and potential rapid escape from mAb binding and neutralization could be some likely reasons for the variation between the viruses. In vitro studies with RRV and human immune serum suggested that ADE may occur with increased infection in macrophages and modulation of the pro-inflammatory response [113,114,115]. One study showed sub-neutralizing levels of CHIKV immune plasma increased infection in myeloid cells and augmented the expression of pro-inflammatory cytokines and type I interferon response [116]. When the immune plasma was administered to mice immediately after CHIKV infection, there was increased foot swelling and viremia [116]. Although these experiments were not repeated in FcγR-deficient mice to confirm Fc-mediated enhanced disease, this phenotype should be considered when evaluating efficacy of mAbs targeting alphaviruses. Neutralizing and non-neutralizing mAbs against EEEV, VEEV, and WEEV have been isolated, characterized, and shown to be protective against aerosol and subcutaneous challenge in mice and non-human primates [117,118,119,120,121]. Some of the most protective mAbs in vivo were either mouse IgG2a/c or human IgG1 and/or non-neutralizing, which suggests that Fc effector functions may be at least partially involved [117,118,121]. Future work should determine if Fc-FcγR interactions are necessary for mAb therapy against the encephalitic alphaviruses. While this is not trivial as virulent strains of EEEV and VEEV are select agents and the encephalitic viruses require a BSL3 lab, the optimal mAb activity should be analyzed for potential therapies.

### 3.4. Flaviviruses

Flaviviruses are enveloped viruses with a single-stranded, positive sense RNA genome in the *Flaviviridae* family and are primarily transmitted by arthropods. Some significant viruses of interest in this genus include Zika virus (ZIKV), West Nile virus (WNV), dengue virus (DENV), and yellow fever virus (YFV). Infection with flaviviruses can produce a range of symptoms from asymptomatic, mild, or moderate illness to severe disease including encephalitis, shock, liver failure, or congenital malformations depending on the virus [122]. The 2015–2016 ZIKV epidemic in Central and South America illustrated the continued emerging threat of flaviviruses and the need for new therapeutics [4]. Flaviviruses attach and enter cells through binding of the E protein, which is also the main target of neutralizing antibodies on the virion [122]. Sub-neutralizing levels of antibodies targeting proteins on the virion can result in ADE, which enhances infection in an FcγR-dependent manner in myeloid cells and can be prevented using antibodies that lack Fc effector functions [8,122]. For the purposes of this review, we will not discuss anti-E antibodies and ADE, but rather antibodies directed to the NS1 protein, which is not present in the virion but is expressed in the cell as a monomer, where it is required for virus replication, on the cell surface as a dimer, and secreted as a hexamer [123]. The NS1 protein has three distinct domains: a hydrophobic β-roll, wing domain, and β-ladder [123]. The secreted form of NS1 can be detected in plasma during flavivirus infection, albeit to different levels depending on the virus, and has been shown to have immunomodulatory activities, such as engaging TLR4, interacting with complement factors, inducing autoantibodies, and increasing vascular permeability [124,125,126]. Antibodies targeting the NS1 can reduce the activity of secreted NS1 and enhance clearance of infected cells through Fc-FcγR interaction and complement activation through binding of the surface bound NS1.

Human and murine mAbs targeting the NS1 of ZIKV, WNV, YFV, and DENV have been isolated, characterized, and shown to be efficacious in animal challenge models. One group isolated four human anti-ZIKV NS1 mAbs, all of which were IgG1, that bound to ZIKV infected cells, engaged FcγRIIIa and activated NK cells in vitro [127]. Administration of one of the mAbs, AA12, to *Stat2^−/−^* adult mice increased survival and reduced weight loss, clinical disease, and viral burden in spleens following ZIKV challenge [127]. When the AA12 LALA or LALA-PG variant was administered to mice, protection was lost [127]. Since ZIKV has been linked to fetal malformations including microcephaly, the protection of NS1 mAbs during pregnancy is a critical focus. A panel of mouse and human anti-ZIKV NS1 mAbs was characterized and a subset reduced viral titers in the spleens and brains of adult mice with the mouse Stat2 knocked out and replaced with the human STAT2 (STAT2-KI) [15]. LALA variants of the human mAbs were generated and failed to reduce viral burden in the brain of adult STAT2-KI mice [15]. The protective mAbs mapped to the wing or β-ladder platform domain of the NS1 protein [15]. When these mAbs were tested in pregnant STAT2-KI dams, a single mouse mAb, Z17, reduced viral load in the placenta and fetal head, and a combination treatment with two mouse mAbs, Z15 and Z17, further reduced the viral titers [15]. Treatment with a human mAb, 749-A4, reduced virus in the placenta and fetal head in an Fc-dependent manner [15]. In vitro Z17 and 749-A4 enhanced ADCP in neutrophils and monocytes, and increased complement deposition, suggesting that in vivo protection during pregnancy is potentially mediated by one of these mechanisms [15]. Another study addressed the protective efficacy of human anti-ZIKV NS1 mAbs in neonate mice. Multiple administrations of mAbs targeting the N-terminal region (β-roll), 4B8 and 3G2, or the C-terminal region (β-ladder), 4F10, of NS1 significantly increased weight gain and survival and 4B8, and 3G2 reduced viral burden in the brain [128]. Importantly, mAb treatment resulted in higher brain mass, decreased lymphocyte infiltration, and reduced neurological score [128]. All of the mAbs induced ADCC in vitro, but only the 4F10-LALA-PG variant showed reduced protection during in vivo challenge compared to the LALA-PG variants of 4B8 and 3G2 [128]. In vitro analysis showed 4B8 and 3G2 could reduce release of virions when added at late times post-infection, suggesting a potential alternative mechanism of protection [128].

In agreement with the mAb studies, a modified vaccinia Ankara vectored vaccine expressing the ZIKV NS1 completely protected mice from a lethal challenge and reduced dissemination into the brain [129]. As expected, the serum from vaccinated mice failed to neutralize ZIKV but engaged FcγRIIIA in an ADCC assay and induced complement mediated lysis in vitro [129]. In addition to an antibody response, the vaccine induced a strong CD8^+^ T cell response [129]. Although passive or adoptive transfer studies were not performed to identify the main correlate of protection, it is anticipated that the antibody response mediated some level of protection. A DNA vaccine expressing the ZIKV NS1 protein followed by two boosts of adjuvanted recombinant ZIKV NS1 induced high titers of anti-NS1 antibodies that engaged FcγRIV using in vitro assays [130]. Passive transfer of immune serum increased survival and reduced clinical disease following a lethal ZIKV challenge in *Stat2^−/−^* mice with the prototype ZIKV strain, MR766, or a contemporary strain from the 2015–2016 outbreak in the Caribbean indicating that anti-NS1 antibodies are the main correlate of protection for this vaccine [130].

Anti-NS1 mAbs for other flaviviruses have also been characterized. A protective, non-neutralizing, IgG2a anti-YFV NS1 mAb, 1A5, bound to NS1 on the cell surface, protected mice from lethal infection, and blocked YFV replication in the brain [60,131]. F(ab’)_2_ variants of 1A5 did not block replication in the brain [60]. When the mAb was isotype-switched to IgG1 or a mixture of IgG1 and IgG2b, the mAbs failed to reduce viral load in the brain and reduced survival following YFV challenge [60]. Depletion of “killer cells” with cyclophosphamide treatment resulted in loss of protection from lethality with 1A5 treatment, indicating that Fc-FcγR interaction with immune cells was critical for mAb-based protection [60]. A panel of mouse anti-WNV NS1 mAbs was isolated and a subset of mAbs provided greater than 70% survival following lethal challenge compared to 17% in PBS-treated controls [132]. One of the mAbs, 17NS1, reduced viral load in the peripheral tissues, which ultimately reduced the spread of the virus to the central nervous system [132]. When FcRγ^−/−^ mice were treated with 17NS1, there was a loss in protection against lethal infection [132], while another mAb, 14NS1, still protected in FcRγ^−/−^ mice and C1q^−/−^ mice [132]. Both mAbs were IgG2a and bound surface expressed NS1 in vitro, so this dichotomy could be related rather to the epitope of the mAbs [132,133]. Survival with 10NS1 administration was also FcγR-dependent, specifically FcγRI or IV, and not mediated by NK cells, which predominated express FcγRIII [133]. Based on in vitro assays with peritoneal macrophages, 10NS1 enhanced phagocytosis in an FcγRI or IV-dependent fashion [133]. This indicates that at least one mechanism of protection in vivo with the anti-WNV NS1 mAbs could be through ADCP. DENV anti-NS1 mAbs have been shown to reduce the pathogenic effects of DENV NS1 such as blocking endothelial dysfunction and vascular leakage, and reducing mast cell degranulation [134,135,136]. For some of these studies, the protective efficacy of the anti-NS1 mAbs was not Fc-dependent [134,135].

ADE is a constant concern for the development of flavivirus vaccines and mAb therapeutics, but using antibodies that target proteins not associated with the virion eliminates this risk. The above studies demonstrate the effectiveness of mAbs targeting viral proteins that are present in the cell rather than on the virion. While these mAbs are non-neutralizing, many of them protect in vivo through Fc effector functions. However, unlike antibodies targeting the E glycoprotein, antibodies targeting the NS1 protein will never provide sterilizing immunity. The anti-NS1 epitopes may be a factor in the necessity of Fc-FcγR or Fc-complement interaction, while other epitopes protect using an alternative mechanism that reduces release of the virus from infected cells. A powerful therapeutic strategy would be to pair NS1 antibodies with those targeting the E glycoprotein with a modified Fc region, which would combine the Fc functionality with potential sterilizing immunity. The ZIKV research using pregnant mice demonstrates a role for mAb treatment in the prevention of virus-induced congenital disorders, and Fc-FcγR interaction appears to be an essential for this protection.

### 3.5. Filoviruses

Filoviruses are a taxonomic family (*Filoviridae*) of enveloped viruses with a single-stranded, negative sense RNA genome. Viruses in this family cause severe disease in humans and are responsible for sporadic, regional outbreaks of viral hemorrhagic fever. The filoviruses of greatest concern are part of two taxonomic genera, *ebolavirus* and *marburgvirus*, which are commonly referred to as Ebola virus (EBOV) and Marburg virus (MARV) [137]. The viral envelope of these viruses is studded with homotrimeric glycoproteins (GP) that undergo proteolytic cleavage by host proteases into two subunits, GP1 and GP2, which mediate attachment to the host cell and fusion with the host membrane, respectively [138]. Although no single receptor has been identified for any of the filoviruses, virus attachment is thought to be mediated by a highly glycosylated region of the GP1 subunit designated the mucin-like domain (MLD) [139,140]. EBOV and MARV infections have high case fatality rates, and few licensed treatments exist for these diseases, which makes them ideal candidates for the development of mAb therapies. The EBOV epidemic in Western Africa in 2014–2016 provided renewed urgency and interest in the development of passive immunization treatments, and a large number of mAbs were isolated and described in the years following this outbreak [141,142,143,144,145,146].

The contribution of Fc-mediated functions in the efficacy of anti-EBOV mAbs had been implicated by the early observation that neutralization efficiency of anti-GP antibodies was not always predictive of in vivo efficacy [147,148,149,150,151]. The systematic comparison of large (>150) panels of human and mouse anti-EBOV antibodies using profiling pipelines involving neutralization assays, in vitro Fc effector function assays, in vivo therapeutic models, and machine learning analysis confirmed that neutralization is not the only determinant of the efficacy of anti-EBOV mAbs and that Fc-mediated phagocytosis and NK cell activation were strongly correlated with protection [145,146]. Antibodies that were protective had varying combinations of neutralization activity and Fc-effector functionality, and these different combinations resulted in similar efficacies [145]. These profiling studies provide strong support for a role of Fc-FcγR interaction in mAb treatment of EBOV but are limited by the lack of experiments specifically focused on characterizing Fc contributions in vivo. Bournazos et al. (2019) evaluated the role of Fc function in neutralizing antibodies targeting different epitopes of the EBOV GP using in vivo challenge models. Specifically, the study included antibodies targeting the MLD, the interface region between the GP1 head and the glycan cap (chalice bowl), the fusion loop domain on GP2, and the stalk region of the GP (HR2 domain and MPER region) [14]. Intact versions of each antibody were compared to recombinant versions with both diminished (GRLR) and enhanced (GASDALIE) FcγR affinities in a lethal EBOV challenge of humanized FcγR mice [14]. The Fc-FcγR interaction was dispensable for the antibodies targeting the MLD, HR2 domain, and MPER region but was indispensable for the antibodies targeting the chalice bowl and fusion loop [14]. Earlier work comparing the in vivo protection of the anti-MPER mAb BDBV223 to BDBV223-LALA found Fc function to be necessary for efficacy [152]. These differences in Fc functionality between two antibodies targeting the MPER region reinforces the presumption that the contribution of Fc function towards therapeutic antibody protection is specific to each antibody and not generalizable to antibodies targeting the same domain or epitope. The mouse mAb m8C4 provided therapeutic protection in lethal mouse models of multiple divergent EBOVs and is one of the first mAbs to show pan-EBOV efficacy [153]. Evaluation of Fc function of m8C4 indicates high phagocytic activity in monocytes, macrophages, neutrophils, and DCs compared to other murine anti-EBOV mAbs, which hints at a role for Fc function in cross-filovirus protection [153]. MR228 is a non-neutralizing anti-MARV mAb targeting the GP2 portion of the MLD that provides therapeutic protection in a lethal challenge of MARV in both mice and guinea pigs [154]. When Fc function is ablated by introduction of the LALA mutations, the therapeutic protection of MR228 is lost in mice, but impairment of Fc-C1q interaction by introduction of the KA mutation had no effect on efficacy in mice [154]. In addition to the loss of protection, treatment with MR228-LALA resulted in higher viral titers in spleen, blood and lymph nodes compared to the intact antibody [154]. In guinea pigs, loss of Fc function did not abolish protection but did result in higher viral titers [154]. The differences between the two models may indicate species-specific differences in Fc functionality during viral infections between mice and guinea pigs or could be a spurious discrepancy due to the complexity of the two models.

The status of filoviruses as both a risk group 4 pathogen and a pathogen of unique concern (i.e., select agent, category A pathogen, etc.) limits the number of laboratories capable of studying these viruses and by extension limits the breadth of research. Despite these inherent difficulties, many anti-filovirus mAbs have been characterized and much is known about the mechanisms underlying their in vivo efficacy. This work has already spawned the first USDA-approved therapeutic against Zaire ebolavirus with the antibody cocktail Inmazeb [155]. Based on the summarized work, broad conclusions about the role of effector function can be drawn. Neutralization activity should not be used as the sole determinant of antibody prioritization or selection. Fc-mediated functions are important contributors to the in vivo efficacy of many anti-filovirus antibodies, with the primary cellular mediators being phagocytic cells (monocytes, macrophages and DCs). The systemic comparison of large panels of anti-EBOV antibodies demonstrates that the contribution of neutralization or effector function towards overall efficacy are not fixed biological concepts, and the contribution of each mechanism varies across mAbs. This observation could be very important for the selection of mAbs for antibody cocktails, as the inclusion of mAbs with both strong neutralization and effector function may yield better therapeutics.

## 4. Conclusions

Emerging viral infections are a serious threat to global public health. As the human population continues to grow and expand into formerly non-domesticated habitats, the risk of zoonotic spillover, as evidenced by the COVID-19 pandemic, and range expansion of endemic viruses, as evidenced by the 2015 Zika virus epidemic, grows more consequential every year. In the face of these epidemic and pandemic risks, there is a need for therapeutics that can be developed quickly after the discovery of emerging viral infections. To that point, mAbs are an effective therapeutic strategy for the treatment of viral infections and are ideally suited for the treatment of emerging infections. Monoclonal antibodies can be rapidly isolated from convalescent patients or produced in laboratory animals, and they have a good safety profile with limited adverse reactions reported in human trials [156]. Additionally, multiple pan-family neutralizing antibodies have been described, opening the possibility for treatments targeting entire viral families including yet-to-be discovered viruses. Neutralization has long been thought to be the principal mechanism of action of antibodies during viral infections but as demonstrated by the studies highlighted in this review, Fc-mediated effector functions play a significant role in the antibody response to viral infections. The importance of Fc-FcyR interactions varies between antibodies and may be influenced by epitope location, binding affinity, breadth of reactivity, neutralization potency, and time of administration. While the contribution of specific Fc-mediated functions varies depending on the antibody being tested and the experimental model, our synthesis of these studies demonstrates a significant role for phagocytic cells. Reduced in vivo efficacy was observed in multiple viral systems with the depletion of phagocytic cells including monocytes and macrophages (Table 2). In addition to their role as mediators of ADCP and ADCC, the recent work with SARS-CoV-2 supports a role for these cells in skewing the immune response either towards a pro-inflammatory response or a more beneficial tissue repair and homeostasis response [11]. Critically, this immunomodulation can be influenced by treatment with a particular mAb (Figure 1).

Considering antibody functions beyond neutralization should be part of the early screening process for the identification of mAbs that are worthy of further evaluation and development. The antibody screening pipelines discussed in the Filovirus section offer a potential blueprint for evaluating large panels of mAbs, but any future studies should also include in vivo testing of antibodies as no reliable surrogate exists for live animal studies [145,146]. The concerns about ADE have long tempered the clinical application of mAbs against certain viral infections. While these concerns are legitimate, particularly for some flaviviruses, research has demonstrated that targeting proteins not present on the virion eliminates this risk but still retains the therapeutic efficacy. The proteins present on the virion surface are often important targets for mAb therapy, but characterizing and developing mAbs targeting other viral proteins should also be pursued. The majority of mAbs are administered intraperitoneal (in animals) or intravenous (in animals and humans) to achieve systemic distribution, but these routes of administration offer limited protection at mucosal surfaces. As emerging respiratory viruses are a major public health concern, alternative routes of administration, including intranasal delivery, are an important area for future research. The intrinsic biological properties of IgG make this isotype the predominant focus of antibody-based therapeutics, but improvements in half-life and kinetics of other isotypes could lead to therapeutics tailored to the site of infection as would be the case with IgA and respiratory or enteric infections [157]. An additional application of mAbs is towards prevention of infection through prophylactic treatment of at-risk populations. This approach is the focus of at least one clinical trial to prevent COVID-19 in high risk populations, such as nursing homes or regions of uncontrolled outbreaks (ClinicalTrials.gov Identifier: NCT04452318) [158]. Mouse models are an invaluable tool for evaluating the role of antibody effector functions, but they lack the genetic variation of the FcγR that is observed in humans. The influence of FcγR diversity on the efficacy of mAbs is beyond the scope of this review but is an emerging area of research that could have significant impact on the widespread use of these therapeutics [159,160]. Most importantly, creating antibody cocktails with mixed Fab and Fc functionality could aid in greater control of viral infections and reduce the likelihood of viral escape. Optimization of Fc-FcγR interactions will produce better mAb therapies and aid in reducing unnecessary mortality due to emerging viral infections.

## Figures and Tables

**Table 1:** Summary of signaling function, mouse and human IgG interactions, and cell type expression of mouse FcγRs.

Mouse FcγR	Function	Mouse IgG Interactions	Human IgG Interactions	Cellular Expression in Mice
FcγRI	Activation	IgG2a/c IgG2b IgG3 ^#^	IgG1 IgG3 IgG4	dendritic cells macrophages monocytes
FcγRIIb	Inhibition	IgG1 IgG2b IgG2a/c	IgG1 IgG2 IgG3 IgG4	neutrophils B cells dendritic cells macrophages monocytes
FcγRIII	Activation	IgG1 IgG2a/c IgG2b	IgG1 IgG2 IgG3 IgG4	NK cells neutrophils dendritic cells macrophages monocytes
FcγRIV	Activation	IgG2a/c IgG2b	IgG1 IgG3 IgG4 *	neutrophils dendritic cells macrophages monocytes

^#^ Debated. * Weak binding.

**Table 2:** Summary of in vivo depletion or adoptive transfer studies to identify cell types involved in Ab-mediated protection.

Virus	Antibody (Species)	Neut ^+^	Identified Cell Type (Depletion or Transfer Method)	Outcome ^$^	Ref **
Influenza A virus	2B9, 2C10, FEE8 (mouse)	No	AMΦ * [clodronate liposomes or adoptive transfer of AMΦ (2B9)]	Depletion- increased weight loss, viral load at 6 dpi, and mortality	[56]
				Transfer- reduced weight loss and increased survival	
	5E01, 5D06 (human)	No	AMΦ (clodronate liposomes)	Depletion- increased weight loss, mortality	[56]
	9H10, 6F12 (mouse)	Yes	AMΦ (clodronate liposomes)	Depletion with suboptimal mAb dose- increased weight loss	[56]
	FI6v3 (optimized human)	Yes	CD8^+^ T cells (anti-CD8)	GAALIE or GA variant plus depletion- increased weight loss and mortality.	[35]
	M2e-HBc immunized	No	NK and NKT cells (anti-asialo-GM1)	Depletion- increased mortality	[57]
	M2e-HBc immune serum (passive transfer)	No	AMΦ * (clodronate liposomes or adoptive transfer of AMΦ)	Depletion- increased weight loss and mortality	[58]
				Transfer- reduced weight loss and increased survival	
SARS-CoV-2	COV2-2050 (human)	Yes	Ly6C^hi^ monocytes (anti-CCR2)	Depletion- increased weight loss, lung pathology, and cytokines and chemokines	[11]
			CD8^+^ T cells (anti-CD8)	Depletion- increased viral burden at 8dpi in lungs	
SARS-CoV	Immune serum	Yes	AMΦ and monocytes (clodronate liposomes and/or anti-Gr1)	Depletion- increased viral titer in lungs at 9 dpi. Highest titer when both AMΦ and monocytes were depleted	[59]
Chikungunya virus	CHK-152 + CHK-166 (mouse & humanized)	Yes	Monocytes (anti-CCR2)	Depletion- increased viral burden in ankle at 7 dpi and increased foot swelling	[12]
Yellow Fever virus	1A5	No	“killer cells” (cyclophosphamide treatment)	Depletion- reduced survival	[60]
West Nile virus	E28	No	Macrophages (clodronate liposomes)	Depletion- reduced viremia 1 and 2 dpi	[61]

^+^ Neut = Neutralizing. Determined using in vitro assays. ^$^ Outcome indicates the phenotype when the specified cell type was depleted or transferred with anti-viral antibody treatment compared to administering anti-viral antibody or cell transfer alone. ** Ref = Reference. * AMΦ = alveolar macrophage.
